# Fever, fear and hunger: the response of the Irish population to infectious disease during the Great Irish Famine, 1845-48

**DOI:** 10.1186/1753-6561-6-S4-O23

**Published:** 2012-07-09

**Authors:** R McDermott

**Affiliations:** 1 University of Newcastle, UK

## Introduction

Over one million people died during the Great Irish Famine. Around one third of these perished as a result of infectious disease. The aim of this study is to analyse the reaction of the Irish people to the ‘fever’, a term used at the time, combining typhus, typhoid fever and relapsing fever, and cholera epidemics that swept through the country during the famine years. Previous historical scholarship has focussed on the medical establishment. This study moves beyond that to analyse the reaction of the sufferers themselves.

## Methods

The basis of the study is a rigorous analysis of the famine folklore collected in the 1930s and 1940s by the Irish Folklore Commission. This is combined with a study of sources from the period, both government records and the writings of those who visited the worst hit areas.

## Results

The folklore provided a strikingly different perspective on infectious diseases during the famine. Food was believed to be the most significant cause of sickness, in contrast to modern medical thought on the causes of these infections. Notions of contagion were well understood at the time, and produced much panic among the population. This panic had a profound effect on strongly held cultural beliefs. The most surprising result was the response of the medical establishment. In contrast to previous scholarship, this work argues that physicians and the hospital system were peripheral to the care given to the people, with the clergy providing the bulk of the assistance the sick needed.

## Conclusions

The reaction of the Irish people to infectious disease was complex, consisting of many different theories. These theories had a significant effect on the way the population actually dealt with ‘fever’ and cholera. This study is an important part of the on-going project to construct a social history of medicine in Ireland. It also shows the utility of folklore in allowing the researcher a perspective on a group whose beliefs and reactions are unobtainable from conventional sources.

